# ID 81 - Evidências Científicas na Tomada de Decisão: uma reflexão para organizações de saúde

**DOI:** 10.5327/2237-9622.2025.v34s1.81

**Published:** 2025-11-25

**Authors:** Rachel Riera, Silvio Fernandes da SIlva, Romeu Gomes, Maria Lúcia Teixeira Machado, Jorge Otávio Maia Barreto, Tereza Setsuko Toma

**Keywords:** Políticas Informadas por Evidências, organizações de saúde, tomada de decisões, Sistema Único de Saúde

## Abstract

**Introdução:**

O uso das melhores evidências disponíveis para a tomada de decisão em saúde é fundamental para qualificar a utilização dos recursos públicos, propiciando eficiência, eficácia, efetividade, equidade e transparência na formulação, implementação e monitoramento das políticas. As Políticas Informadas por Evidências (PIE) orientam a escolha das melhores opções para abordar problemas prioritários de saúde pública. O objetivo do estudo foi analisar os resultados do projeto Apoio à Formulação e Implementação de Políticas de Saúde Informadas por Evidências 2015-2023.

**Método:**

O estudo utilizou a triangulação entre os resultados do projeto, a Teoria de Tomada de Decisão de Simon e a experiência dos autores. A análise baseou-se na questão: Quais avanços, limites e aprendizados foram observados na implementação do uso de evidências científicas na tomada de decisão em contextos organizacionais?

**Resultados:**

Duas estratégias foram desenvolvidas no projeto: (1) capacitação de pessoas em gestão de PIE e (2) elaboração de ferramentas para ampliar o uso de PIE. As capacitações ocorreram por meio de três cursos com o objetivo de desenvolver capacidades cognitivas, individuais e em equipe e apoiar os educandos na aplicação dos conhecimentos em suas organizações (mais de 90% públicas). Os cursos utilizaram uma abordagem construtivista e um processo de formação apoiado territorialmente por docentes/facilitadores. Essas iniciativas ocorreram em 36 cidades de todas as regiões geográficas do Brasil, além de duas turmas fora do País. No período 2021-2023, em alinhamento com as diretrizes do planejamento estratégico do Departamento de Ciência e Tecnologia do Ministério da Saúde e com a Rede EVIPNet, o projeto desenvolveu ferramentas com potencial de sensibilizar e apoiar os envolvidos no processo decisório: https://docs.bvsalud.org/biblioref/2023/11/1517423/guia-para-implementar-nev.pdf; e Perfil de Competência do Profissional em PIE no Brasil – https://docs.bvsalud.org/biblioref/2023/04/1427485/espie-perfil-competencia-pie.pdf.

**Conclusão:**

A articulação entre decisores de diferentes estações terapêuticas para promover um cuidado integrado foi considerada nos planos de intervenção, a partir do entendimento de que o cuidado perpassa os níveis organizacionais e, muitas vezes, a própria organização. O papel das autoridades no sentido de legitimar novos conhecimentos também esteve presente nas estratégias de disseminação de PIE. O envolvimento de gestores, por exemplo, os secretários de saúde, foi considerado como um importante fator de sucesso em PIE. Com relação aos aspectos que competem com a apropriação de novos conhecimentos, foram colocadas em prática estratégias de mapeamento da motivação de atores relevantes para a viabilização das intervenções e de convencimento para que aderissem às medidas propostas. Constatou-se que a incorporação de conhecimentos nas organizações de saúde é um processo complexo que precisa considerar dinamicidade da cadeia decisória, comportamento das autoridades no sentido de favorecê-los ou legitimá-los e barreiras arraigadas na cultura da organização e que competem com novos conhecimentos.

